# Evolution of membrane signaling and trafficking in plants

**DOI:** 10.3389/fpls.2013.00040

**Published:** 2013-03-05

**Authors:** Markus Geisler

**Affiliations:** Plant Biology – geislerLab, Department of Biology, University of FribourgFribourg, Switzerland

**Figure 1:**
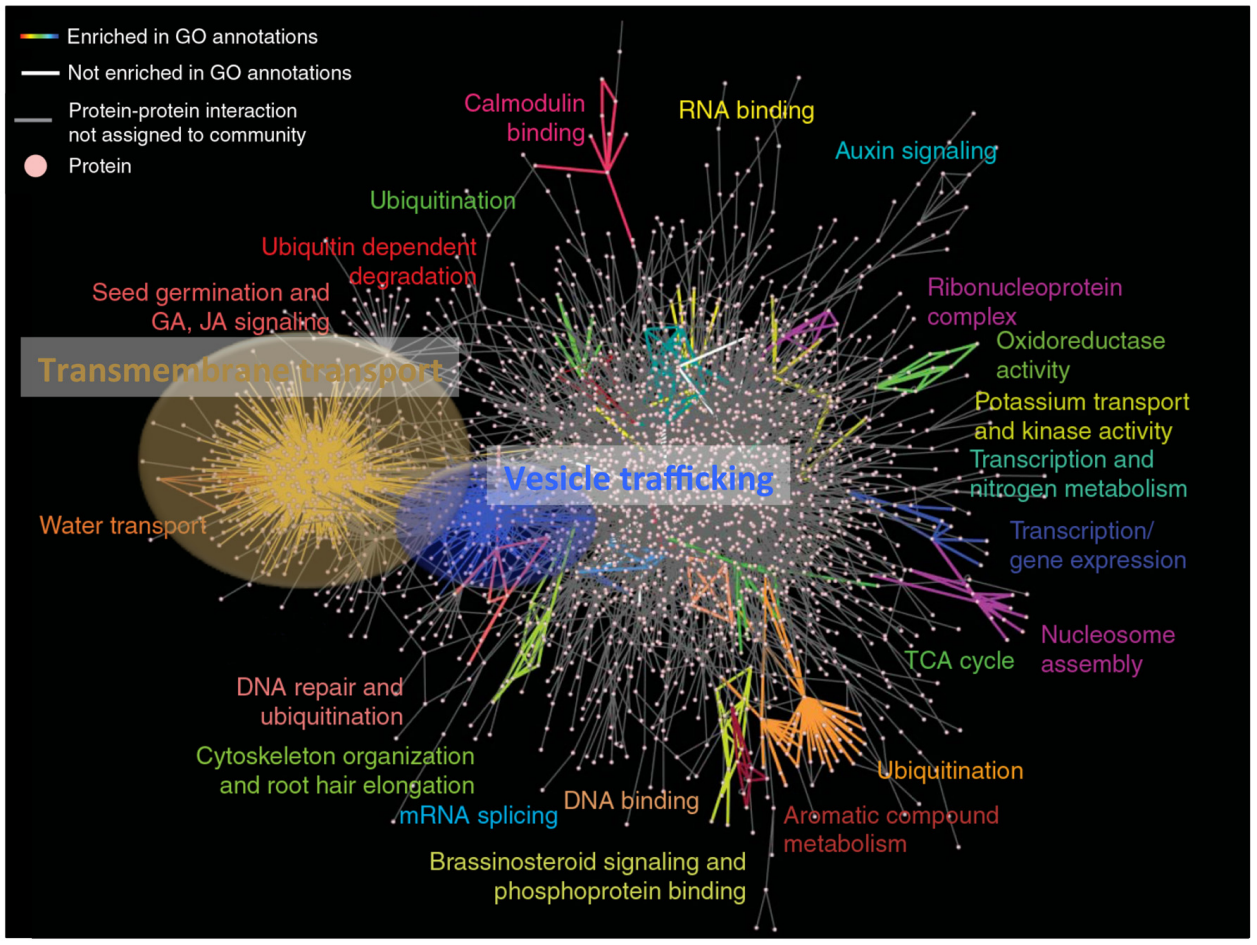
**The large transmembrane transport community (brown) shares a high amount of proteins with the vesicle trafficking community (blue) suggesting a strong physical and functional overlap and interaction**. AI-1_MAIN_ communities enriched in gene ontology annotations are colored. Modified from Arabidopsis Interactome Mapping Consortium (2011).

Membrane proteins are essential determinants of many biological processes in plants. They function in metabolic processes, signal transduction, transport of small molecules and polymers across endo- and plasma membranes, and inter-compartmental trafficking of proteins, lipids, and cell wall components. During these highly integrative processes, dynamic interactions of membrane proteins with other membrane or soluble components are thought to provide a high degree of flexibility that usually characterizes higher plants. This concept is supported by the recent release of a first, partial Arabidopsis interactome by the Arabidopsis Interactome Mapping Consortium [http://interactome.dfci.harvard.edu/A_thaliana; (Arabidopsis Interactome Mapping Consortium, 2011)]. The Arabidopsis interactome reveals a strong enrichment of a few network communities, with the transmembrane transport community being the largest. Strikingly, the high degree of shared proteins among the transmembrane transport and trafficking communities suggests both physical interaction and functional overlap (see Figure 1).

With no surprise, this current research topic co-hosted by *Frontiers in Plant Transport and Traffic* and *Frontiers in Plant Physiology* reflects well this outcome of the *Arabidopsis* Interactome Mapping Consortium. It embraces outstanding current research efforts with a series of reviews and original papers that highlight conserved and plant-specific mechanisms of post-Golgi trafficking, exocytosis and endocytosis and the role of the actin cytoskeleton from an evolutionary perspective. This research topic is comprised of reviews on PILS and AUX/LAX transmembrane auxin carriers and reviews, original articles, and hypothesis and theory articles on plant channels, including potassium channels, slow and quick anion channels, and glutamate receptors. This research topic concludes with an integrated view on how membranes shape plant nodulation and Arbuscular mycorrhizae.

As such, this research topic is a timely update presenting advances in research in the evolution of membrane signaling and trafficking in plants, encompassed of outstanding contributions that address fundamental questions in these essential processes in plants.
